# Interferon-stimulated gene 15 induces cancer cell death by suppressing the NF-κB signaling pathway

**DOI:** 10.18632/oncotarget.12160

**Published:** 2016-09-21

**Authors:** Hongwu Mao, Man Wang, Biyin Cao, Haibin Zhou, Zubin Zhang, Xinliang Mao

**Affiliations:** ^1^ Jiangsu Key Laboratory for Translational Research and Therapeutics of Neuro-Psycho-Diseases, Department of Pharmacology, College of Pharmaceutical Sciences, Soochow University, Suzhou, Jiangsu, P.R. China; ^2^ Department of Orthopedics, The Second Affiliated Hospital, Soochow University, Suzhou, P.R. China; ^3^ Jiangsu Key Laboratory of Preventive and Translational Medicine for Geriatric Diseases, Soochow University, Suzhou, China

**Keywords:** ISG15, NF-κB, apoptosis, cancer

## Abstract

Interferon-stimulated gene 15 (ISG15) is an important cytokine that has been reported in carcinogenesis. However, we found that ISG15 and de-ISGylase USP18 were induced by several anti-cancer agents, which was confirmed by both RT-PCR and immunoblotting assays. Further studies demonstrated that ectopic ISG15 and USP18 inhibited proliferation of myeloma, leukemia and cervical cancer cells. More importantly, ISG15 and USP18 induced cancer cell apoptosis. This finding was confirmed in a cervical xenograft model in which cervical cancer growth was suppressed by lentiviral ISG15. In the mechanistic study, ISG15 was found to disrupt the NF-κB signaling pathway by downregulating the expression of IKKβ and p65, phosphorylation of p65 and IκBα. Consistent with this finding, ISG15 suppressed the expression of NF-κB recognition element-driving luciferase and decreased the transcription of XIAP and Mcl-1, two typical genes regulated by NF-κB. Therefore, the present study demonstrated that ISG15 induces cancer cell apoptosis by disrupting the NF-κB signaling pathway. This study highlighted a novel role of ISG15 in tumor suppression.

## INTRODUCTION

Interferon-stimulated gene 15 (ISG15) is a cytokine secreted from monocytes and lymphocytes upon infection or the treatment of Type I interferon (IFN-α/β) [1]. In addition, ISG15 is the first reported ubiquitin-like modifier of various proteins. As a modifier, ISG15 could be covalently attached to specific substrate proteins to modulate their biological functions. This modification is called ISGylation in a manner similar to protein ubiquitination which is directed by a series of distinct enzymes including ISG15 activating enzyme (E1, such as UBE1L), the ISG15 conjugating enzyme (E2, such as UBCH8), ISG15 ligases (E3, such as HERC5 and HERC6), and ISG15-specific proteases such as USP18 (or UBP43) [1].

In the last decade, ISG15 has been frequently reported in various cancer tissues including hepatocellular carcinoma [2], pancreatic cancers [3], colorectal cancer [4]. For example, ISG15 is differentially expressed in nasopharyngeal carcinoma and is correlated with local recurrence and short overall survival and disease-free survival [5]. Therefore, ISG15 is regarded as a cancer promoter. However, several other studies show that ISG15 also displays anti-cancer activities [6, 7], for example, it affects the cancer microenviroment and inhibits cancer progression [8].

In the present study, we found that ISG15 was induced by anti-cancer agents clioquinol (CLQ) [9] and mefloquinine (MFQ) [10], and more importantly, ISG15 is able to trigger cancer cell apoptosis by modulating the NF-κB signlaing transduction.

## RESULTS

### ISG15 and USP18 were upregulated by anti-cancer agents

**Figure 1:**
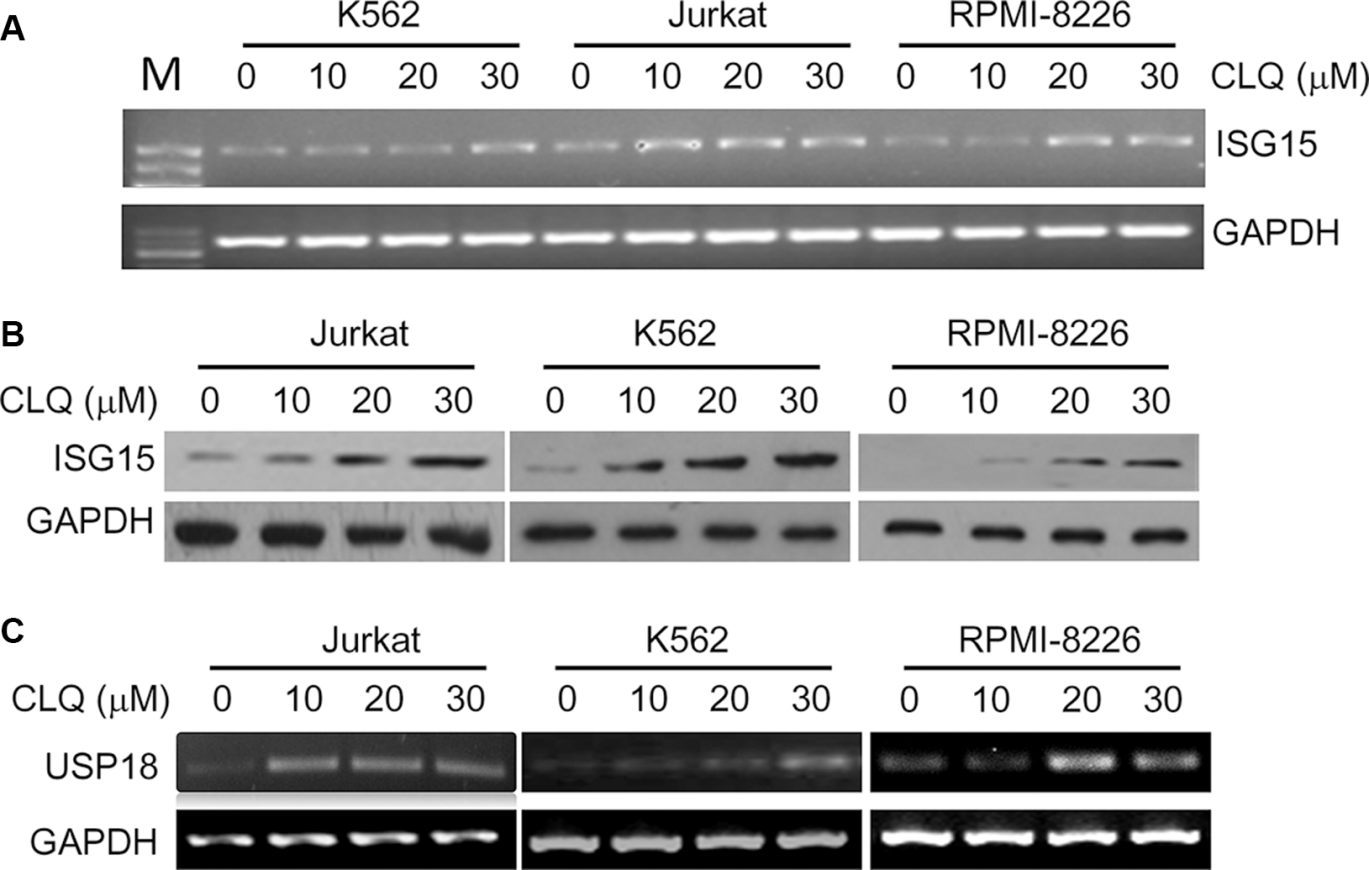
ISG15 and USP18 are induced by clioquinol (CLQ) (**A**) Jurkat, K562 and RPMI-8226 cells were treated with CLQ at indicated concentrations for 24 hrs followed by total RNA preparation. ISG15 mRNA expression was measured by RT-PCR. M: DNA Marker. (**B**) Total protein extracts were prepared from (A) cells, followed by immunoblotting against ISG15. (**C**) USP18 mRNA was measured from (A) samples by RT-PCR.

CLQ and MFQ are two recently identified anti-leukemia agents, to find out the effects of CLQ and MFQ on gene expression associated with their anti-cancer activity, DNA microarray analyses were performed in OCI-AML2, a typical acute myelogenous leukemia cell line. To our surprise, ISG15, the first reported ubiquitin-like small protein, was induced by both CLQ and MFQ in a time-dependent manner. As shown in Table 1, ISG15 was induced more than 13 and 8 folds within 24 hrs by CLQ and MFQ, respectively. In addition, the de-ISGylation enzyme USP18 was also upregulated by CLQ and MFQ. As shown in Table 2, USP18 was upregulated 4.2 and 6.9 folds by CLQ at 24 and 30 hrs, respectively, and it was also upregulated for 3.19 and 4.67 folds by MFQ at 24 and 48 hrs, respectively. To verify these effects, the lymphoma cell line Jurkat, leukemia cell line K562 and myeloma cell line RPMI-8226 were treated for 24 hrs by CLQ at increasing concentrations, both RT-PCR and immunoblotting analyses demonstrated that ISG15 was induced in a concentration-dependent manner (Figure 1A and 1B). Moreover, USP18 was also induced in these cell lines by CLQ dependent on the increased concentrations (Figure 1C). These results thus indicated that ISG15 and USP18 were induced by anti-cancer agents in blood cancer cells. Notably, these results were consistent with previous studies [11, 12], in which ISG15 was significantly induced by classic anti-cancer camptothecin [11] and all-trans retinoic acid [12].

**Table 1:** ISG15 was induced by MFQ and CLQ in AML cells

Treatment	Genes	Probes	Fold Changes	Regulation
CLQ-30 hrs	ISG15	205483_s_at	13.42	up
MFQ-48 hrs	ISG15	205483_s_at	8.74	up

**Table 2:** USP18 was induced by MFQ and CLQ in a time-dependent

Drugs	24 hrs	30 hrs	48 hrs	Regulation
CLQ	4.256	6.949	-	up
MFQ	3.193	-	4.674	up

### ISG15 suppresses cancer cell proliferation

**Figure 2:**
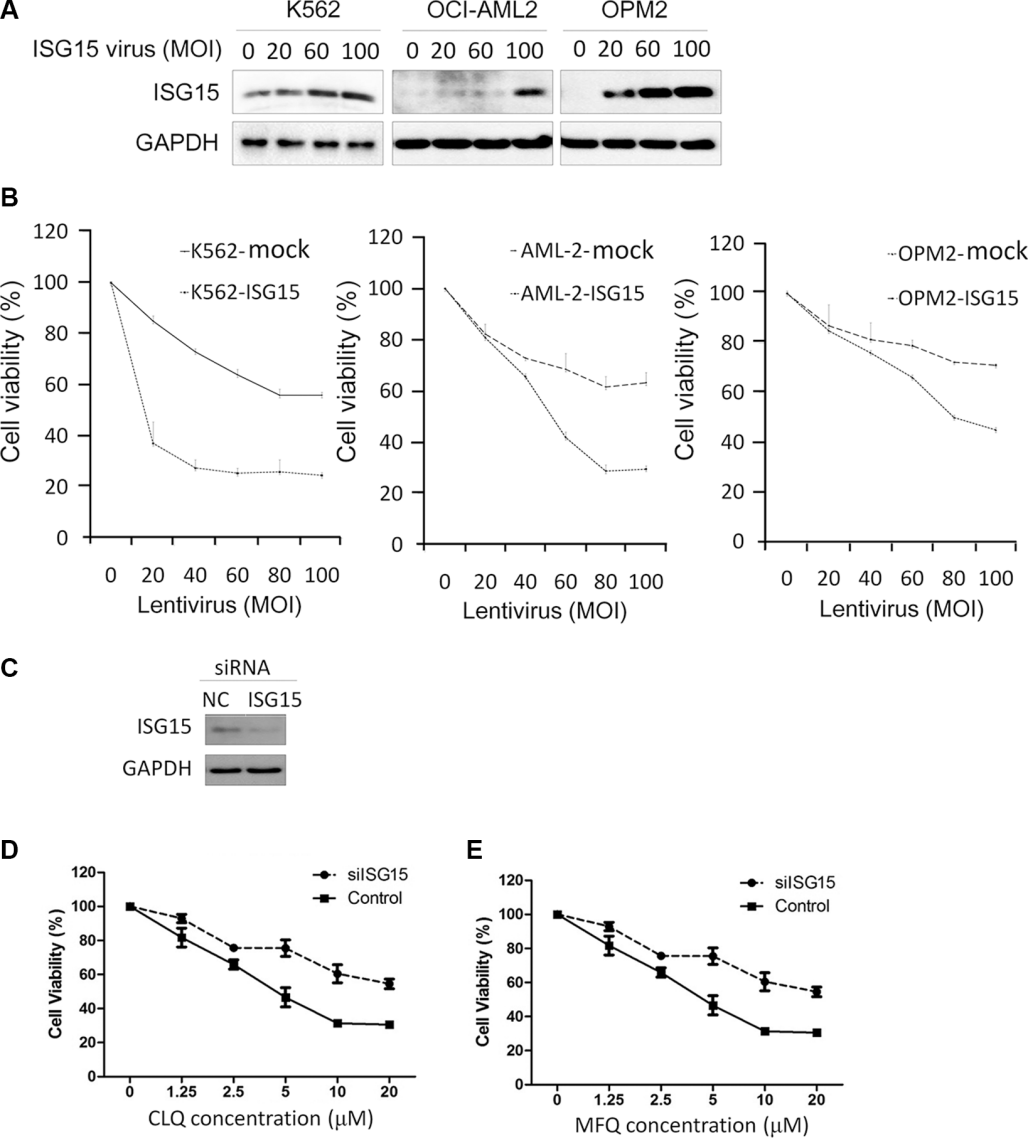
ISG15 suppresses cancer cell proliferation (**A**) K562, OCI-AML2 and OPM2 cells were infected with lentiviral ISG15 for 96 hrs, followed by immunoblotting assay to evaluate ISG15 expression. (**B**) The above cells infected with lentiviral ISG15 or mock lentivirus for 96 hrs were subjected to cell viability analysis by MTT. (**C**) K562 cells were transfected with siISG15 and incubated for 48 hrs, followed by immunoblotting assay to evaluate ISG15 knockdown. (**D**, **E**) K562 cells transfected with siISG15 were subjected to CLQ (D) or MFQ (E) treatment for 72 hrs followed by MTT assay for cell proliferation.

Because ISG15 was upregulated by anti-cancer agents, we wondered whether it could suppress cancer cell proliferation. To this end, K562, OCI-AML2, and OPM2 were infected with ISG15 lentivirus followed by cell proliferation assay. As shown in Figure 2A, ISG15 expression was increased in cells with the increase of MOI (multiplicity of infection). Notably, ISG15 suppressed proliferation of all three cell lines in a MOI-dependent manner (Figure 2B). To further understand the role of ISG15 in anti-cancer action, K562 cells were transfected with siISG15 (Figure 2C) followed by CLQ and MFQ treatment. As shown in Figure 2D and 2E, knockdown of ISG15 markedly increased cell resistance to both CLQ and MFQ. Therefore, these results further demonstrated that ISG15 could suppress cancer cell proliferation.

### Ectopic ISG15 and USP18 induce cancer cell apoptosis

**Figure 3:**
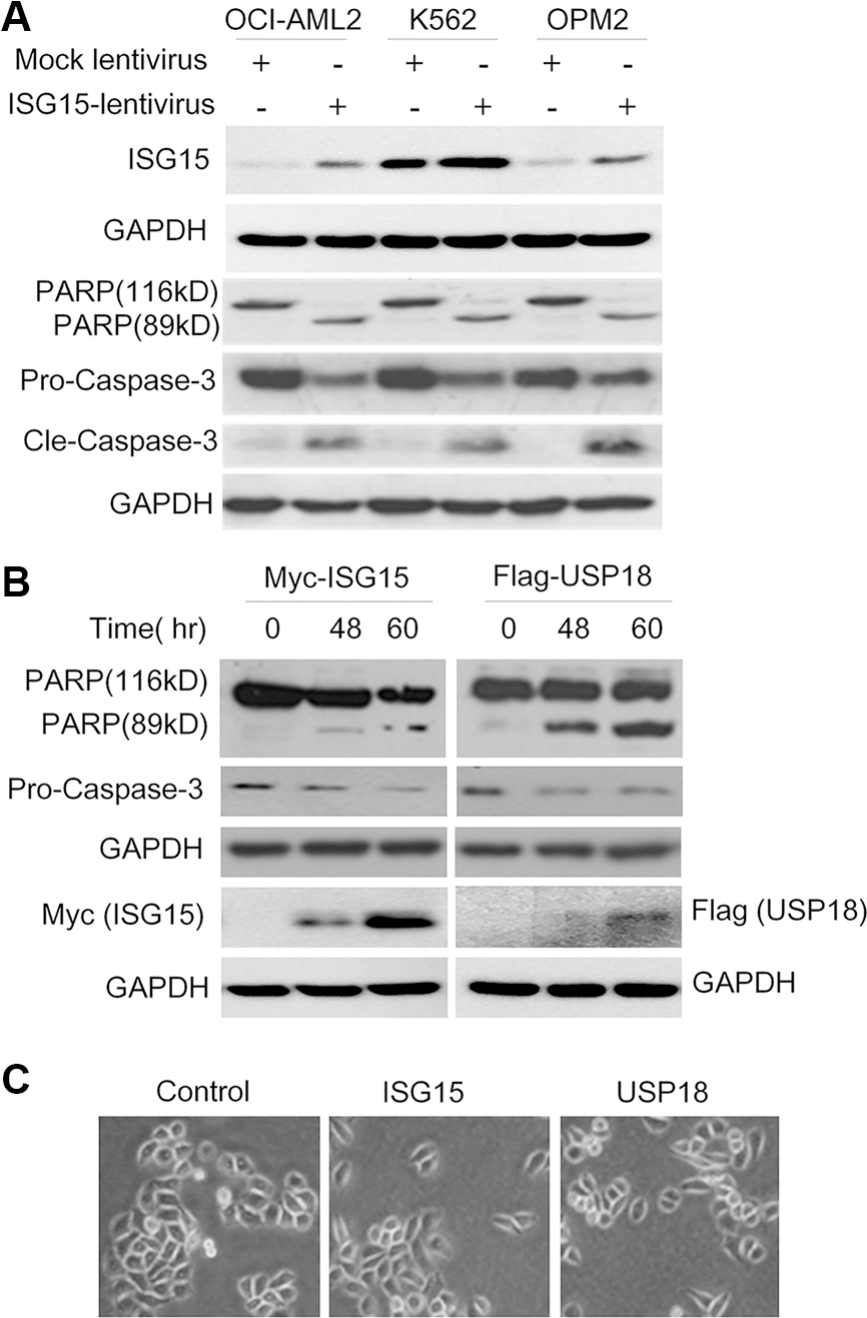
Ectopic ISG15 and USP18 induces cancer cell apoptosis (**A**) Blood cancer cells were stably infected with lentiviral ISG15 or mock lentivirus. Cell lysates were harvested at 96 hrs after infection to measure PARP and Caspase-3 cleavage by immunoblotting assay. (**B**) HeLa cells were transfected with ISG15 or USP18 plasmids for 48 or 60 hrs before being collected for immunoblotting against PARP and Caspase-3. (**C**) HeLa cells were transfected with ISG15 and USP18 plasmids, respectively. Cell proliferation was analyzed on a microscope and the cell images were photographed at 48 hrs after transfection.

The above studies implicated that ISG15 suppressed cancer cell proliferation, to find out whether it induced cancer cell apoptosis, we evaluated apoptosis in leukemia and myeloma cells by overexpressing ISG15. As shown in Figure 3A, lentiviral ISG15 led to the cleavage of both PARP and Caspase-3 in all cell lines examined, suggesting the apoptotic signaling was triggered. To confirm this effect, HeLa cells were transfected with ISG15 and USP18 plasmids, respectively, with increasing concentrations or extended incubation time. Consistent with the results in leukemia and myeloma cells, both PARP and caspase-3 were cleaved by ISG15 and USP18 in HeLa cells (Figure 3B). To further evaluate the effects of ISG15 and USP18 on HeLa cell proliferation, HeLa cells transfected with ISG15 or USP18 plasmids were analyzed under a microscopy. As shown in Figure 3C, ISG15 and USP18 individually suppressed HeLa cell proliferation. Therefore, all the above studies demonstrated that ISG15 and USP18 alone induced apoptosis in leukemia, myeloma and cervical cancer cells.

### ISG15 delays tumor growth *in vivo*

**Figure 4:**
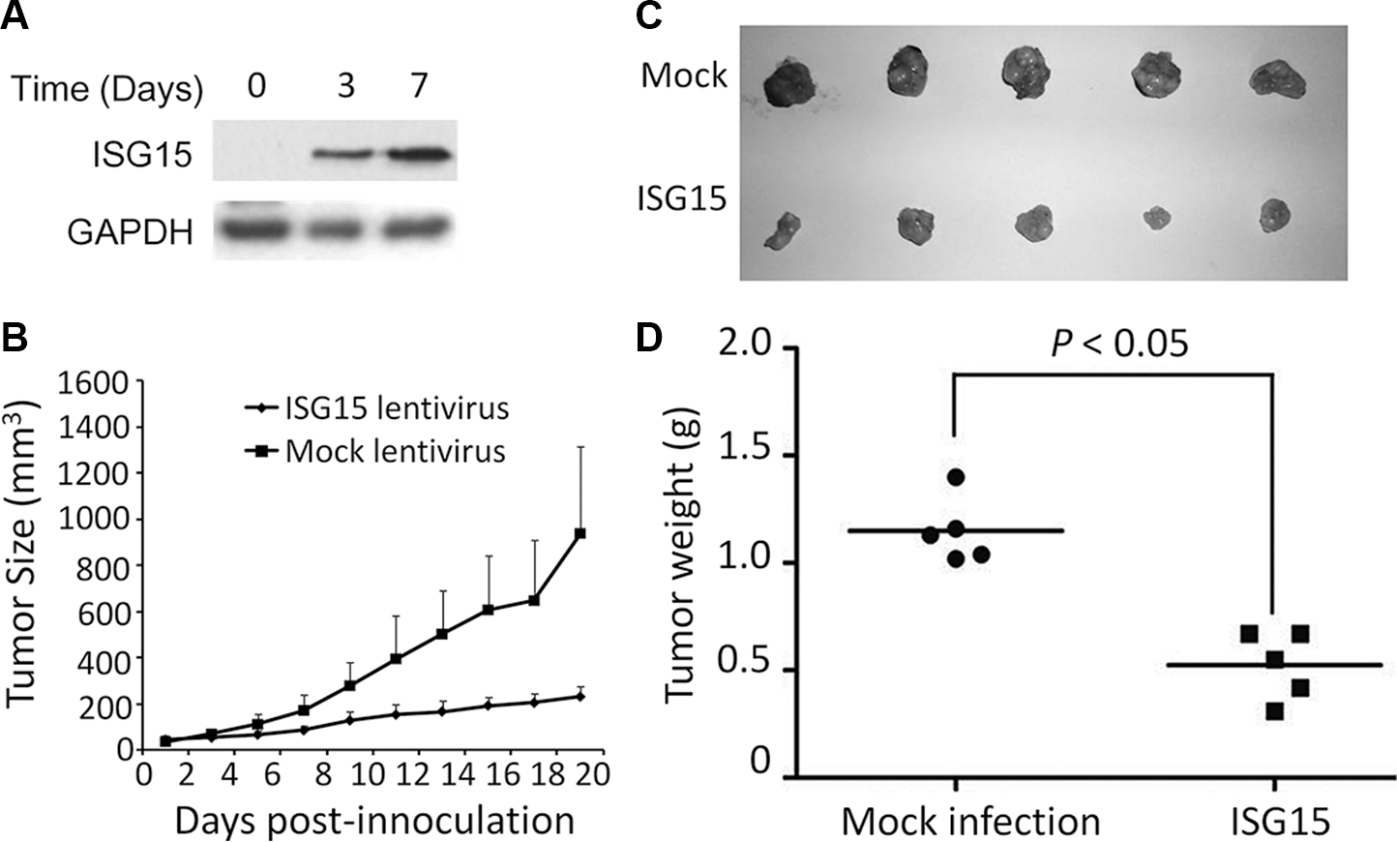
ISG15 suppresses cervical cancer growth *in vivo* (**A**) HeLa cells were infected lentiviral ISG15, the ISG15 level was measured at Day 3 and 7 before being innoculated into nude mice. (**B**) Female nude mice (BALB/c) at 5–6 weeks old were innoculated with HeLa cells infected with lentiviral ISG15 or empty virus. When tumor sizes were measured from the next day (as Day 1) for 19 days. (**C)** Tumors were obtained at the last day of the experiment. (**D**) tumor tissues were subjected to weight analysis.

To evaluate the anti-cancer activity of ISG15 *in vivo*, we next established a human cancer xenograft model in nude mice using HeLa cells which were infected with lentiviral ISG15 or empty virus (Figure 4A). Tumor growth was monitored over 19 days. As shown in Figure 4B, the growth rate of the tumors from ISG15-expressing HeLa cells were significantly slower than the mock control (Figure 4B). At the end of the experiment, the tumor sizes from ISG15-expressing HeLa cells were markedly smaller than those of the mock ones (Figure 4C) and the average tumor weight was significantly smaller in ISG15-expressing group (Figure 4D). Therefore, ISG15 displayed anti-tumor activity *in vivo*.

### ISG15 inhibits NF-κB signal transduction

**Figure 5:**
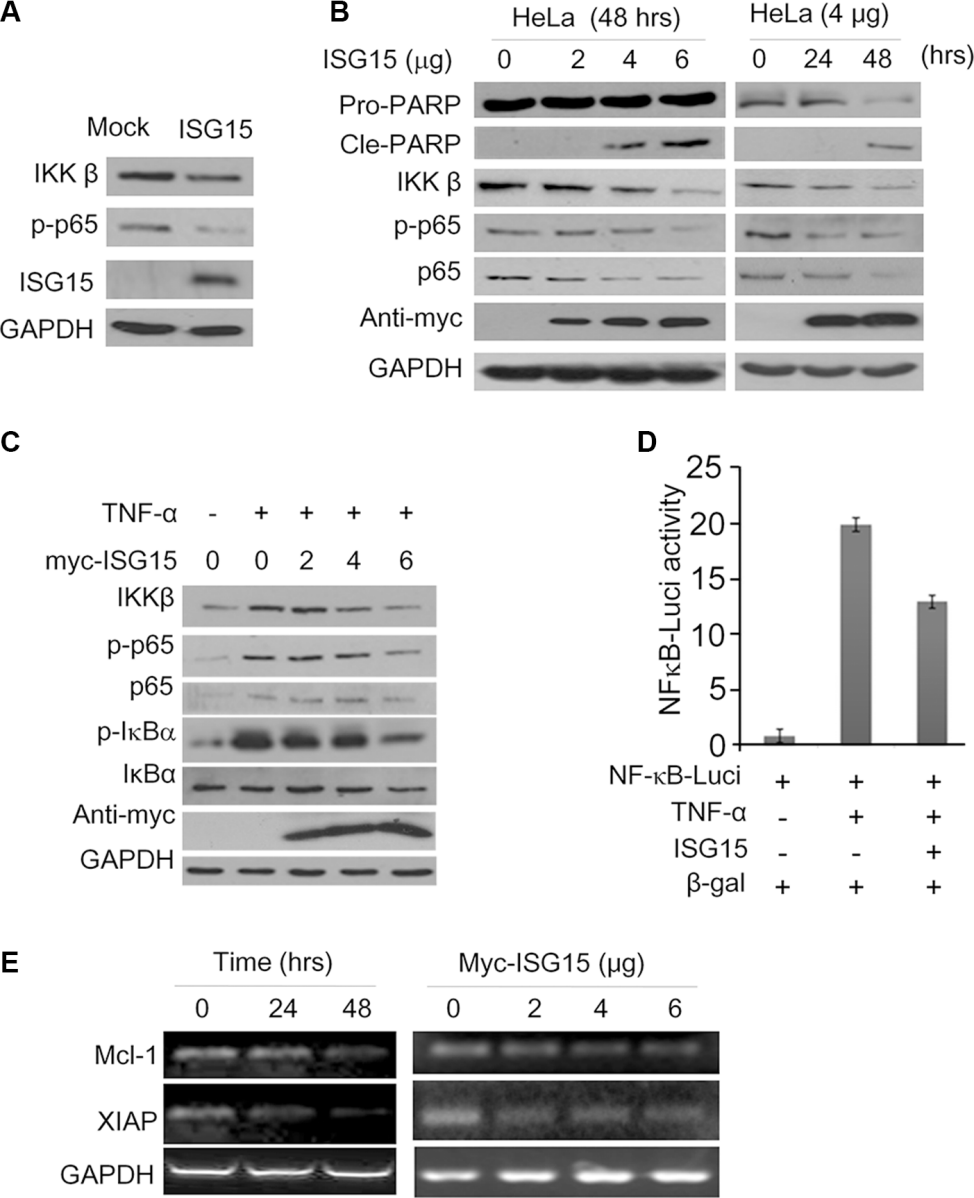
ISG15 inhibits the NF-κB signaling transduction (**A**) K562 cells were infected with lentiviral ISG15 for 96 hrs followed by immunoblotting assay against indicated proteins using specific antibodies. (**B**) HeLa cells were transfected with ISG15 plasmids at indicated concentrations and time periods. Cells were harvested to measure the NF-κB signaling components and PARP. (**C**) After being transfected with ISG15 at indicated concentrations for 48 hrs, HeLa cells were starved overnight (at 0.5% serum), followed by TNF-α (100 ng/mL) treatment for 30 min before being subjected to immunoblotting assay against specific proteins as indicated. (**D**) HEK293T cells were transfected with NF-κB-Luci and or ISG15 plasmid for 24 hrs, followed by TNF-α (100 ng/mL) treatment for 30 min. Cell lysates were then prepared for luciferase assay. (**E**) HeLa cells were transfected with ISG15 at indicated concentrations for 24 or 48 hrs. The mRNA levels of Mcl-1 and XIAP were evaluated by RT-PCR.

The above studies indicated that ISG15 alone displayed anti-cancer activity by inducing cancer cell apoptosis and inhibiting cell proliferation. Because ISG15 is regarded as a cytokine in response to interferon treatment in association with the NF-κB signaling, a key player in carcinogenesis, we wondered whether NF- κB was involved in the ISG15-mediated cell apoptosis. To this end, K562 cells were infected with ISG15 lentivirus followed by immunoblotting assays. As shown in Figure 5A, when ISG15 was introduced, IKKβ and p-p65, two of the key components in the NF-κB signaling pathway, were decreased. Subsequently, we evaluated it in HeLa cells. After transfected with ISG15 plasmids, HeLa cells were subjected to immunoblotting assay against specific proteins in the NF-κB signaling pathway. It turned out that ISG15 downregulated the NF-κB signaling in a time- and concentration-dependent manner. Consistent with the findings in K562 cells, ISG15 downregulated the expression of IKKβ, p-p65, and p65 in HeLa cells, along with PARP cleavage that indicated cells underwent apoptosis (Figure 5B). We next measured this effect of ISG15 in starved HeLa cells treated with TNF-α, a key cytokine stimulating the NF-κB signaling pathway. As expected, TNF-α activated the NF-κB cell signaling by upregulating IKKβ expression and increasing the phosphorylation of p65 and IκBα, but these signals were suppressed by ISG15 in a concentration-dependent manner (Figure 5C). To further convince this finding, we evaluated NF-κB activity using firefly luciferase as the reporter under control of NF-κB recognition element [13]. As shown in Figure 5D, TNF-α markedly increased the luciferase activity, but it was suppressed by ISG15. Notably, ISG15 also decreased the transcription of anti-apoptotic proteins including Mcl-1 and XIAP, two typical genes modulated by NF-κB (Figure 5E). Therefore, all the above results clearly demonstrated that ISG15 suppressed the NF-κB signal transduction and induced cancer cell apoptosis.

## DISCUSSION

Although it has been proposed in carcinogenesis involved in several types of cancers, including lung cancer, breast cancer, and others, our present study suggests that ISG15 displays anti-cancer activity by disrupting the NF- κB signaling pathway.

As a key player in cancer-association inflammation, there is no doubt that ISG15 is involved in carcinogenesis which has been demonstrated in several lines of investigative evidence. ISG15 is associated with poor prognosis and it is implicated in cytoskeleton disruption and cancer cell migration [14]. Although the detailed mechanisms are lacking, ISG15 modulates pluripotency-associated genes expression, maintains cancer stem cell phenotype, and promotes tumorigenesis [5, 15].

However, ISG15 is probably a double-edged sword in carcinogenesis and cancer suppression. As demonstrated in the present study, both ISG15 and the deIGSlyation enzyme USP18 are upregulated by anti-cancer agents CLQ and MFQ. More importantly, enforced ISG15 leads to cancer cell apoptosis and decreased cancer cell proliferation. These findings is consistent with previous studies in which all-trans-retinoic acid (RA) promotes the transcription of ISG15, USP18, and ISGylation activating enzyme UBE1L in RA-sensitive but not resistant leukemia cells [12]. ISG15 was also induced by classic anti-cancer agent camptothecin [11]. This study along with these previous studies thus suggest that in contrast to promoting tumorigenesis, ISG15 also displays anti-cancer activities. Actually several key proteins have been found involved in ISG15-induced cancer cell apoptosis. For example, ISG15 upregulates the expression and anti-cancer activity of p53 because knockdown of ISG15 decreases p53 ubiquitination and suppresses its activity. ISG15 knockdown also downregulates the transcription of p21, a key tumor suppressor and negative modulator of cell cycle progress [16]. These reported findings partly explain the anti-cancer activity of ISG15. In the present study, we found that ISG15 triggers apoptosis by disrupting the NF-κB signal transduction [17]. ISG15 not only suppresses the expression of p65 and IKKβ, but also inhibits the phosphorylation of p65 and IκBα. Moreover, ISG15 abolishes TNF-α-activated NF-κB signaling and decreases the transcription of NF-κB-modulated genes including XIAP and Mcl-1, two oncogenes in various cancers. This study complements with a previous study that found ISG15 interferes with TRAF-6 polyubiquitination and subsequent downregulation of the NF-κB activity [17]. The present study provided a novel piece of direct evidence that ISG15 disrupts the NF-κB signaling.

It is well known that ISG15 exists in two forms, the free one and conjugated one. In the present study, we found that ISG15 and its deISGylase USP18 were induced by anti-cancer agents, and each alone induces cancer cell apoptosis. This finding is consistent with a recent study in which free ISG15 is found to show potent anti-cancer activity *in vivo* because free ISG15 increases NK cell infiltration into xenografted tumors in nude mice and suppresses tumor growth [6, 8]. However, ISG15 conjugates are also critical. An earlier study showed that ISG15 expression leads to ISGylation and subsequent degradation of oncogenic ΔNp63α, a variant of p63 and a negative modulator of p53 activity, that promotes anchorage-independent cell growth and tumor formation *in vivo* [7]. This is reasonable because ISG15 can lead to ISGylation of various proteins as a protein modifier with high similarity to ubiquitin, ISG15 probably interferes with protein ubiquitination, and alters the biological function of the substrate proteins [7, 16, 17]. Therefore, ISG15 probably displays itself as a double-edged sword in cancer dependent on its free form or conjugation context.

In summary, we provided a novel line of evidence showing that ISG15 is an endogenous tumor suppressor. Taken together with previous findings that ISG15 promotes oncogenesis, it should be cautious when making a conclusion of ISG15 and ISGylation into cancer therapy.

## MATERIALS AND METHODS

### Cell culture

Human blood cancer cells RPMI-8226 cells were cultured in Iscove's modified Dulbecco's medium; Jurkat, OCI-AML2, K562 and HeLa cells were cultured in RPMI-1640 medium; human HEK293T cells were cultured in Dulbecco's high glucose modified Eagle's medium. All media were supplemented with 10% fetal bovine serum (Hyclone).

### Cell proliferation

Cells were infected with lentiviral ISG15 for 96 hrs followed by MTT assay as described previously [13]. To directly analyze cell growth and proliferation of HeLa cells, cell images were photographed using an inverted microscope (Nikon) as described previously [18].

### Plasmids

To clone ISG15 and USP18 and generate individual plasmids, total RNA was extracted using the TRIzol^®^ Reagent (Invitrogen) according to the manufacturer's instructions. cDNA was synthesized from equal quantities of total RNA using the EasyScript First-Strand cDNA Synthesis SuperMix (TransGen Biotechnology). To generate ISG15 plasmid, a forward primer containing a XhoI recognition site (5′-CCCTCGAGATGGAACAAAAACTTATTTCT G-3′) and a reverse primer containing an EcoRI (5′-CGGAATTCTTAGCTCCGCCCGCCAGGC-3′) were designed; to generate USP18 plasmid, a forward primer containing a BamHI recognition site (5′-ACGGGAT CCATGAGCAAGGCGTTTG-3′) and reverse primer containing a XhoI recognition site (5′-TACCGCTC GAGTTAGCACTCCATCTTC-3′) were applied. Both sequences were cloned into a pcDNA3.1 vector with a myc-tag.

### Gene expression studies

OCI-AML2 cells were treated with 10 μM of CLQ or 5 μM of MFQ (All from Sigma-Aldrich) for 24, 30 or 48 hrs before collected for RNA preparation and gene expression profiling analysis according to our previous report [19]. Microarray data were analyzed using GeneSpring GX v10.0 (Agilent), and lists of genes deregulated > 2-fold were subject to further study.

### Preparation of ISG15 lentivirus

The full-length *ISG15* gene was amplified by PCR with primers, forward 5′-CGGAATTCATGGAACAAAA ACTTATTTCTGAA-3′ and reverse 5′-AAGGAAAAAA GCGGCCGCTTAGCTCCGCCCGCCA-3′. The underlined sequences were recognized with EcoR I and Not I, respectively. The ISG15 gene was then inserted between EcoRI and Not I of the pCDH (System Biosciences) or pLVX-puro (Clontech) lentiviral vectors. The viral production was produced with a standard method as manufacturer's instructions including control and package plasmids (Shanghai GeneChem Co., Ltd., Shanghai, China), and these plasmids were co-transfected into HEK293T cells with calcium precipitate method as described previously [20].

### Reverse transcription-polymerase chain reaction (RT-PCR)

RT-PCR was performed in a 25-μL reaction system containing 12.5 μL 2 × Easy TaqSuperMix (TransGen Biotechnology, Beijing, China) as described previously [21]. Specific primers were as below: ISG15 forward 5**′**-TG GACAAATGCGACGAACC-3′ and reverse 5′-TTCGTC GTTCACTCGCC-3′; USP18 forward 5′-CCCACAGGCT CATAACTAAA-3′ and reverse 5′-AATATGAACCATGAG GCCCC-3′; GAPDH forward 5′-AGTCCACTGGCG TCTTCA-3′ and reverse 5′-CTCCGACGCCTGCTTCA CCA-3′.

### Transient transfection

HeLa cells were planted into 10-cm dishes. When in 50% confluence, cells were refreshed with serum-free medium and were subject to transfection with specific plasmids by Lipofectamine 2000 agent (Invitrogen).

### Western blotting analysis

Total proteins (30 μg) were subjected to fractionation on a SDS polyacrylamide gel electrophoresis and immunoblotting assay. Antibodies used in the study included: anti-ISG15, anti-PARP, and anti-Caspase 3 (Cell Signaling Technologies, Cambridge, MA); anti-myc-tag, anti-Flag-tag, and anti-GAPDH (Medical & Biological Laboratories Co., Ltd., Nagoya, Japan); anti-IKKβ, anti-p-p65, anti-p65, anti-IκBα, and anti-p-IκBα (Epitomics, Burlingame, CA); Horseradish peroxidase-conjugate anti-mouse or anti-rabbit secondary antibodies from Beyotime (Nantong, China).

### Luciferase activity assay

A NF-κB luciferase construct was established by cloning a NF-κB recognition element into the pGL4 vector (Promega) as reported previously [13]. Luciferase activity was analyzed using Bright-Gloluciferase assay system as described previously [20].

### Cervical cancer xenograft mice model

Female BALB/c nude mice of 7–8 weeks old were obtained from Shanghai Slac Laboratory Animal Co., Ltd (Shanghai, China) were subcutaneously injected with HeLa cells that infected with ISG15 lentivirus or mock virus. Tumor sizes and mice body weights were measured every other day for 18 days. At the end of the study, tumors were applied for Western blotting against ISG15. The animal experiment was conducted in accordance with the protocols approved by the Review Board of Animal Care and Use of Soochow University.
